# Baseline factors identified for the prediction of good responders in patients with end-stage diffuse coronary artery disease undergoing intracoronary CD34+ cell therapy

**DOI:** 10.1186/s13287-020-01835-z

**Published:** 2020-07-29

**Authors:** Pei-Hsun Sung, Hsin-Ju Chiang, Yi-Chen Li, John Y. Chiang, Chi-Hsiang Chu, Pei-Lin Shao, Fan-Yen Lee, Mel S. Lee, Hon-Kan Yip

**Affiliations:** 1 Division of Cardiology, Department of Internal Medicine, College of Medicine, Kaohsiung Chang Gung Memorial Hospital and Chang Gung University, No. 123, Ta Pei Road, Niao Sung District, Kaohsiung, 83301 Taiwan; 2 Center for Shockwave Medicine and Tissue Engineering, Kaohsiung Chang Gung Memorial Hospital, Kaohsiung, 83301 Taiwan; 3 Department of Obstetrics and Gynecology, College of Medicine, Kaohsiung Chang Gung Memorial Hospital and Chang Gung University, Kaohsiung, 83301 Taiwan; 4 Chung Shan Medical University School of Medicine, Taichung, 40201 Taiwan; 5 Department of Computer Science and Engineering, National Sun Yat-sen University, Kaohsiung, 80424 Taiwan; 6 Department of Healthcare Administration and Medical Informatics, Kaohsiung Medical University, Kaohsiung, 80708 Taiwan; 7 Department of Statistics, National Cheng Kung University, Tainan, 70101 Taiwan; 8 Institute of Statistics, National University of Kaohsiung, Kaohsiung, 80708 Taiwan; 9 Department of Nursing, Asia University, Taichung, 41354 Taiwan; 10 Division of Thoracic and Cardiovascular Surgery, Department of Surgery, Kaohsiung Chang Gung Memorial Hospital and Chang Gung University College of Medicine, Kaohsiung, 83301 Taiwan; 11 Division of Cardiovascular Surgery, Department of Surgery, Tri-Service General Hospital, National Defense Medical Center, Taipei, 11490 Taiwan; 12 Department of Orthopedics, College of Medicine, Kaohsiung Chang Gung Memorial Hospital and Chang Gung University, Kaohsiung, 83301 Taiwan; 13 Institute for Translational Research in Biomedicine, College of Medicine, Kaohsiung Chang Gung Memorial Hospital and Chang Gung University, Kaohsiung, 83301 Taiwan; 14 Department of Medical Research, China Medical University Hospital, China Medical University, Taichung, 40402 Taiwan; 15 Division of Cardiology, Department of Internal Medicine, Xiamen Chang Gung Memorial Hospital, Xiamen, 361028 Fujian China

**Keywords:** CD34+ cell therapy, Good responders, Diffuse coronary artery disease, Refractory angina, Left ventricular ejection fraction

## Abstract

**Background:**

Treating patients with end-stage diffuse coronary artery disease (EnD-CAD) unsuitable for coronary intervention remains a clinical challenge. They usually express refractory angina and have a high risk of mortality. Although growing data have indicated cell therapy is an alternative solution to medical or invasive therapy, there are still lacking useful markers to predict whether heart function will improve in the EnD-CAD patients who underwent circulatory-derived CD34+ cell therapy. By utilizing the baseline variables and results from our previous phase I/II clinical trials, the aim of this study tried to elucidate the variables predictive of the “good response” to CD34+ cell therapy.

**Methods:**

This retrospective study included 38 patients in phase I clinical trial (2011–2014), and 30 patients in phase II clinical trial (2013–2017). These patients were categorized into “good responders” and “non-responders” according to their 1-year improvement of LVEF ≥ 7.0% or < 7.0% after intracoronary CD34+ cell therapy. Univariate and multivariate logistic regression models were performed to identify potential independent predictors of a good responder to cell therapy, followed by Hosmer–Lemeshow (H-L) test for goodness of fit and prediction power.

**Results:**

Among baseline data, multivariate analysis demonstrated that the history of a former smoker was independently predictive of good responders (*p* = 0.006). On the other hand, male gender, the baseline Canadian Cardiovascular Society angina score ≥ 3, and grades of LV diastolic dysfunction ≥ 2 were significantly negative predictors of good responders (all *p* < 0.01). After administration of subcutaneous granulocyte-colony stimulating factor (G-CSF), a higher post-G-CSF neutrophil count in addition to the above four baseline variables also played crucial roles in early prediction of good response to CD34+ cell therapy for EnD-CAD (all *p* < 0.03). The H-L test displayed a good prediction power with sensitivity 83.3%, specificity 85.3%, and accuracy 84.4%.

**Conclusions:**

Using the results of our phase I/II clinical trials, previous smoking habit, female sex, lower grades of angina score, and diastolic dysfunction were identified to be independently predictive of “good response” to CD34+ cell therapy in the patients with EnD-CAD.

**Trial registration:**

This is a retrospective analysis based on phase I (ISRCTN72853206) and II (ISRCTN26002902) clinical trials.

## Background

Despite the state-of-the-art pharmacomodulation [1, 2], mature and skillful techniques in a coronary intervention such as percutaneous coronary intervention (PCI) [3, 4] and coronary artery bypass surgery (CABG) [5], continuous education [6], and renewal of guideline for the treatment of coronary artery disease (CAD) [7], the atherosclerotic cardiovascular disease, especially CAD, remains the leading cause of death worldwide. Accordingly, the treatment of CAD remains regrettably an unmet need currently. In light of the aforementioned observation, scientists and physicians are encouraged to seek some potentially therapeutic strategy for patients with complex or severe diffuse CAD who are not only non-candidates for surgical or percutaneous coronary intervention but also refractory to aggressive medical therapy. In fact, previous studies [8–10] in addition to our research [11, 12] have found that a significant number of patients with so-called end-stage diffuse CAD (EnD-CAD) suffered from a refractory symptom of dyspnea or angina and had rather high adverse clinical events and mortality.

Cell therapies for tissue and organ regeneration, including endothelial progenitor cells (EPCs) and mesenchymal stem cells (MSCs), have been extensively investigated in both animal studies and clinical trials in the past two decades [11–17]. Looking closer at these reports, a majority of these investigations done to date focused on the themes of EPC or MSC therapy in improving the ischemia-related heart dysfunction or associated symptoms [11–16]. Additionally, these reported studies [11–13, 18] have demonstrated cell therapies are attractive and promising with favorable clinical outcomes, including improvements in heart failure (HF) symptoms, angina, and left ventricular (LV) systolic function. Most this research [11–13, 18] found the improvement of LV ejection fraction (LVEF) ≥ 5.0 to 7.0% was considered as a “great responder” after cell therapy for refractory angina or EnD-CAD. Surprisingly, while this consensus of “great response” to cell therapy is widely adopted, factors that can be used for the prediction of LVEF improvement ≥ 5.0 to 7.0% have not yet been fully investigated. This could be attributed to the relatively small sample size in previous clinical trials adversely distorting the statistical significance. Currently, the quality assessment of efficacy and the evaluation of potency play key roles in determining the therapeutic success and acceptable quality of cell products [19, 20]. Early prediction of good or great response to cell-based therapy not only is cost-effective but also provides investigators or participants useful information regarding expected beneficial outcomes.

In fact, the patient number in our phase I and phase II clinical trials was actually relatively small so that a *statistical* significance of outcomes might be affected by the small sample size. Accordingly, this study was designed to identify which baseline or early factors have the potential to predict the great response in LVEF improvement after the circulatory-derived autologous CD34+ cell therapy for the treatment of EnD-CAD by aggregating the datasets of our phase I and II clinical trials.

## Material and methods

### Study population

This is a retrospective study conducted in a tertiary medical center. Data were retrieved from our phase I clinical trial entitled “intra-coronary transfusion of circulation-derived CD34+ cells improves left ventricular function in patients with end-stage diffuse coronary artery disease unsuitable for coronary intervention” (registration number: ISRCTN72853206) [11] and phase II clinical trial entitled “intracoronary injection of autologous CD34+ cells improves 1-year left ventricular systolic function in patients with diffuse coronary artery disease and partially preserved cardiac performance unsuitable for coronary intervention—a randomized, open-label, controlled phase II clinical trial (registration number: ISRCTN26002902) [21].

A total of 38 patients with EnD-CAD receiving intracoronary CD34+ cell therapy in phase I trial between December 2011 and March 2014, and 30 patients undergoing CD34+ cell therapy in phase II trial between December 2013 and November 2017, were selected in the present study. The inclusion and exclusion criteria have been thoroughly described in our clinical trials [11, 12, 21]. The written informed consent was obtained from all participants before enrollment. All variables in phase I/II trials were collected and subjected to detailed analysis.

The data acquisitions during the study period, including the clinical and laboratory parameters and imaging studies such as echocardiographic and coronary angiographic findings, have been approved by the Taiwan Food and Drug Administration (TFDA) (IRB No: 99-3985A [phase I], 1066062944 [phase II]) and the Institutional Review Committee on Human Research at Chang Gung Memorial Hospital (IRB No: 96-1381A [phase I], 201003985A0 [phase II]). Both phase I and II clinical trials were conducted at Kaohsiung Chang Gung Memorial Hospital, a tertiary referral center. Additionally, a long-term 5-year follow-up for phase I study was also permitted to perform in the same institute [12].

### Definition of “good responder” after cell therapy for EnD-CAD

The good responder was defined as a 1-year improvement of LVEF ≥ 7.0% after CD34+ cell therapy for EnD-CAD. A cutoff value of LVEF ≥ 7.0% or < 7% was calculated by the average change of LVEF from baseline to 1-year measurement among all participants in our previous phase I [11, 12] and phase II trials [21]. This cutoff value was also considered reasonable after taking several previous reports [13, 22, 23] as references in which the great responder was defined as the mean improvement of at least 5% in LVEF after cell therapy.

### Definition of end-stage diffuse CAD (EnD-CAD)

The definition of EnD-CAD has been described in detail in our previous phase I clinical trial [11]. Briefly, the EnD-CAD was confirmed by coronary angiographic findings which showed more than or equal to one obstructive CAD with severe diffuse morphological feature (defined as the diffuse lesion ≥ 50.0 mm in lengths, especially in relatively distal portion) or total occlusion of the vessel with an unclarified length of the obstruction and non-candidates for PCI or CABG (i.e., since vessel involvement was too diffuse and the diameter was too small for intervention).

### Retrospective collection of the variables and 1-year clinical follow-up

Enrolled patients who successfully underwent stem cell therapy were followed-up for 1 year in the previous phase I/II studies [11, 21]. The baseline characteristics, laboratory data, bench-work results, and imaging findings of coronary angiography (CAG), transthoracic echocardiography, and cardiac magnetic resonance imaging were retrospectively retrieved from our stem cell research database that entered in computers during the previous phase I and II trials. The clinical and preclinical measures at baseline, after granulocyte colony-stimulating factor (G-CSF) administration but prior to CD34+ cell therapy, as well as 1, 3, 6, 9, and 12 months after cell delivery were collected to ensure a thorough analysis. Each patient was regularly followed up at our outpatient clinic, and the relevant clinical information including presentation of symptoms, presence or absence of adverse clinical events, and drug prescription was recorded by research nurses with case report forms, as well as telephone interviews on an irregular basis.

### Procedure and protocol for CD34+ cell isolation

The procedure and protocol were based on our previous report [11]. In detail, the number of CD34+ cells in the mononuclear cell preparation isolated during leukapheresis was enriched by utilizing a commercially available device [COBE Spectra 6.1 (Terumo BCT, INC.)] at 8:00 a.m. through a double lumen catheter inserted into the right femoral vein.

After a time-interval about four hours, an adequate amount of circulatory-derived CD34+ cells was collected and well prepared for intra-coronary infusion. According to the International Society of Hematotherapy and Grafting Engineering (ISHAGE) Guidelines for CD34+ cell determination with flow cytometric measurement of circulating CD34+ cells, hematological stem cells are characterized by the presence of surface markers CD34high/CD45dim/SSClow that were used to quantify the number of isolated CD34+ cells. The formula for the number of circulation-derived CD34+ cells was: number of CD34+ cells = (percentage of CD34+ cells) × WBC count × 10^3^ × peripheral-blood stem cell (PBSC) volume (mL). The flow cytometric analysis followed the current guidelines of the College of American Pathology with a performance coefficient of variation (CV) < 4.0% (3.4 ± 2.5) (by definition, CV < 10.0% is acceptable).

After finishing the CD34+ collection without any cell culture for the differentiation of CD34, the patients were immediately sent to the cardiac catheterization room for receiving the intra-coronary CD34+ cell injection.

### Laboratory assessment of circulating levels of soluble angiogenesis factors

Circulating levels of vascular endothelial growth factor (VEGF), angiopoietin, epithelial growth factor (EGF), hepatocyte growth factor (HGF), transforming growth factor (TGF)-β, and stromal cell-derived growth factor (SDF)-1α, six indicators of soluble angiogenesis biomarkers were measured by duplicated determination with a commercially available ELISA method (R&D Systems, Minneapolis, MN, USA). Intra-observer variability of the measurements was also assessed, and the mean intra-assay coefficients of variance were all < 4.5%.

### Imaging studies

Cardiac magnetic resonance image (MRI) was performed (i.e., prior to and at 6 months after cell therapy) by a radiologist blinded to the treatment allocation of the patients using the current standard evaluation method. In addition, 2D and 3D transthoracic echocardiography were performed by an experienced cardiologist blinded to the patient grouping. The procedure and protocol of 3D transthoracic echocardiography were previously described [12].

### Circulatory-derived mononuclear cells for EPC culture and Matrigel assay for evaluating angiogenesis

The protocol and procedure of EPC culture and the assessment of angiogenesis were based on our previous report [24]. In brief, mononuclear cells (MNCs) were isolated cells and cultivated in differential endothelial cell culture medium (endothelial cell basal medium-2, Cambrex) with 10% fetal bovine serum (FBS), 50 U/mL penicillin, 50 g/mL streptomycin, and 2 mmol/L l-glutamine (Invitrogen) with vascular endothelial growth factor (VEGF) and basic fibroblast growth factor (10 ng/mL) plated on gelatin-coated tissue culture flasks and incubated at 37 °C with 5% CO_2_ for 21 days. The culture medium was changed every 48 h. By day 21, cells with spindle-shaped and cobblestone-like phenotype typical of endothelial cells were found attached on the plate.

The cells with endothelial cell phenotype were then plated in 96-well plates at 1.0 × 104 cells/well in 150 μL serum-free M199 culture medium mixed with 50 μL cold Matrigel (Chemicon International, Inc. Temecula, CA, USA) for 24 h using passages 3 to 4 EPCs incubated at 37 °C in 5% CO_2_. Three random microscopic images (200×) were taken from each well to count cluster, tube, and network formations and the mean values were derived. Both cumulative and mean tube lengths were calculated by Image-Pro Plus software (Media Cybernetics, Bethesda, MD, USA).

### Cardiopulmonary exercise testing

Cardiopulmonary exercise testing (CPET) was used to objectively assess the patients’ functional capacity. The result of peak oxygen update (peak VO_2_) at maximal exercise was recorded as metabolic equivalents, i.e., the best index of aerobic capacity and cardiorespiratory function.

### Statistical analysis

All variables are expressed as mean ± standard deviation or number with a percentage. Independent *t* and Mann-Whitney *U* tests were used to compare the difference in continuous variables between two groups as appropriate. For categorical variables between groups, the variables were compared with chi-square analysis with Fisher’s exact test. Logistic regression models with univariate and multivariate analyses were performed to identify potential independent predictors of a good responder to cell therapy, followed by Hosmer–Lemeshow (H-L) test for goodness of fit in the logistic regression model. In addition, those variables with *p* value < 0.08 in univariate analysis were chosen into multivariate analysis for adjustment. Finally, a nomogram was drawn based on the identified predictors to facilitate the calculation of the probability rate of good response to cell therapy. Statistical analysis was performed using SPSS statistical software for Windows version 22 (SPSS for Windows, version 22; SPSS, IL, USA). A *p* value < 0.05 was considered statistically significant.

## Results

### Baseline characteristics among the EnD-CAD patients (Table 1)

Among 68 subjects receiving CD34+ cell therapy, three patients in the phase II trial were excluded, including one case died of brain stem hemorrhage at 1 month, another one was expired of traumatic cervical spine injury with hypoxia at 1 month, and the other case refused to continue follow-up 1 week after cell delivery. A total of 65 study patients followed up for at least 6 months were selected for the analyses. All EnD-CAD patients expressed high-risk baseline profiles such as 100% for one of the atherosclerotic risk factors, > 76% for diabetes mellitus, > 90% for hypertension, > 87% for dyslipidemia, 70% for PCI history, 100% for multi-vessel CAD, and > 50% for chronic total occlusion (CTO) at left anterior descending (LAD) artery. The age, gender, and rates of body mass index, old stroke, old myocardial infarction (MI), and CABG did not differ between 30 responders and 35 non-responders.

**Table 1:** Baseline characteristics and variables at enrollment

Variable	All (*N* = 65)	Responder (*N* = 30)	Non-responder (*N* = 35)	*P* value
Clinical feature				
Age, year	64.51 ± 8.33	65.47 ± 8.86	63.69 ± 7.89	0.395
Male sex, *n* (%)	52 (80.0%)	21 (70.0%)	31 (88.6%)	0.062
Body height (cm)	161.95 ± 9.97	162.4 ± 7.8	161.7 ± 11.6	0.781
Body weight (kg)	69.68 ± 10.93	70.02 ± 11.96	69.38 ± 10.13	0.814
Body mass index (kg/m^2^)	26.65 ± 4.36	26.50 ± 3.71	26.77 ± 4.89	0.990
Former smoker, *n* (%)	26 (40.0%)	16 (53.3%)	10 (28.6%)	0.042
Hypertension, *n* (%)	59 (90.8%)	26 (86.7%)	33 (94.3%)	0.403
Diabetes mellitus, *n* (%)	50 (76.9%)	23 (76.7%)	27 (77.1%)	0.964
Dyslipidemia, *n* (%)	57 (87.7%)	25 (83.3%)	32 (91.4%)	0.455
Old stroke, *n* (%)	26 (23.1%)	8 (26.7%)	7 (20.0%)	0.525
Old myocardial infarction, *n* (%)	16 (24.6%)	5 (16.7%)	11 (31.4%)	0.168
Chronic hepatitis B or C, *n* (%)	5 (7.7%)	1 (3.3%)	4 (11.4%)	0.363
History of CABG, *n* (%)	22 (33.8%)	7 (23.3%)	15 (49.2%)	0.097
History of PCI, *n* (%)	45 (69.2%)	21 (70.0%)	24 (68.6%)	0.901
Left main involvement, *n* (%)	19 (29.2%)	7 (23.3%)	12 (34.3%)	0.333
Multi-vessel CAD, *n* (%)	68 (100%)	40 (100%)	28 (100%)	1.000
CTO at LAD, *n* (%)	35 (53.8%)	16 (53.3%)	19 (54.3%)	0.939
Laboratory data				
Leukocyte count, 1000/μL	7.00 ± 2.00	6.50 ± 2.07	7.44 ± 1.86	0.017
Hemoglobin, g/dL	13.24 ± 1.87	13.14 ± 2.04	13.32 ± 1.73	0.707
Platelet count, 1000/μL	201.88 ± 57.11	193.1 ± 44.3	209.4 ± 65.9	0.254
Serum creatinine, mg/dL	1.28 ± 0.52	1.25 ± 0.56	1.31 ± 0.48	0.116
eGFR, ml/min/1.73 m^2^	61.33 ± 21.53	62.19 ± 22.73	60.60 ± 20.74	0.770
Serum sodium, mEq/L	140.11 ± 2.69	140.30 ± 2.89	139.94 ± 2.54	0.531
Serum potassium, mEq/L	4.28 ± 0.42	4.35 ± 0.40	4.22 ± 0.44	0.231
Alanine aminotransferase, U/L	27.02 ± 23.33	27.67 ± 28.88	26.46 ± 17.68	0.594
Total cholesterol, mg/dL	163.49 ± 42.01	167.03 ± 36.86	160.46 ± 26.30	0.378
Low-density lipoprotein, mg/dL	96.23 ± 36.34	99.03 ± 34.73	93.83 ± 38.00	0.569
High-density lipoprotein, mg/dL	43.74 ± 9.79	43.97 ± 11.29	43.54 ± 8.45	0.863
Triglyceride, mg/dL	143.20 ± 79.20	132.13 ± 49.16	152.69 ± 97.71	0.989
Medication				
Antiplatelet, *n* (%)	65 (100.0%)	30 (100.0%)	35 (100.0%)	1.000
Anticoagulant, *n* (%)	2 (3.1%)	0 (0.0%)	2 (5.7%)	0.495
Beta-blocker, *n* (%)	58 (89.2%)	20 (100.0%)	28 (80.0%)	0.013
RAS inhibitor, *n* (%)	54 (83.1%)	23 (76.7%)	31 (88.6%)	0.202
Calcium channel blocker, *n* (%)	31 (47.7%)	12 (40.0%)	19 (54.3%)	0.250
Diuretic, *n* (%)	22 (33.8%)	6 (20.0%)	16 (45.7%)	0.029
Lipid lowering agent, *n* (%)	45 (69.2%)	20 (66.7%)	25 (71.4%)	0.678
Vasodilator, *n* (%)	2 (3.1%)	0 (0.0%)	2 (5.7%)	0.495

Notes: Responder was defined as 1-year improvement of LVEF ≥ 7.0% after cell-based therapy for EnD-CAD. Data are expressed as mean ± standard deviation or number (percentage). *Abbreviation*: *CABG* coronary artery bypass grafting surgery, *PCI* percutaneous coronary intervention, *CAD* coronary artery disease, *CTO* chronic total occlusion, *LAD* left anterior descending artery, *eGFR* estimated glomerular filtration rate, *RAS* renin-angiotensin system

The laboratory finding showed that the white blood cell count was significantly lower in responders than in non-responders. However, the platelet count, hemoglobin, estimated glomerulus filtration rate (eGFR), and serum creatinine, total cholesterol, high-density lipoprotein, low-density lipoprotein, and triglyceride did not differ between the two groups.

In addition, responders had a significantly higher prescription of beta-blocker and lower diuretic use than the non-responders. All patients took antithrombotic agents and a majority of them received guideline-directed medical therapy.

### Laboratory parameters after G-CSF injection, clinical presentations, and results of examinations during follow-up, and 1-year outcomes (Table 2)

The post-G-CSF laboratory findings demonstrated that the circulating levels of leukocyte count, hematopoietic stem cells, young cells, neutrophils, CD34+ cells, CD45+ cells, and stem cell percentage measured by flow cytometric analysis, as well as troponin-I did not differ between the two groups.

**Table 2:** Variables within follow-up period and clinical outcomes

Variable	All (*N* = 65)	Responder (*N* = 30)	Non-responder (*N* = 35)	*P* value
Post-GCSF biomarkers				
Leukocyte count, 1000/μL	42.29 ± 10.76	39.49 ± 10.34	44.69 ± 10.67	0.051
Hematopoietic progenitor cell, μL	49.78 ± 46.68	37.00 ± 29.01	59.56 ± 55.05	0.105
Young cell, %	7.21 ± 5.06	7.10 ± 4.93	7.30 ± 5.23	0.859
Segment, %	77.14 ± 7.86	76.86 ± 7.60	77.39 ± 8.18	0.790
Neutrophil count, 1000/μL	32.76 ± 9.31	30.66 ± 9.44	34.56 ± 8.94	0.093
Flow data: stem cell, %	0.35 ± 0.24	0.30 ± 0.18	0.40 ± 0.28	0.226
Performance CV, %	4.68 ± 2.67	4.46 ± 2.71	4.87 ± 2.65	0.540
CD34+ cell, 1000/μL	1.27 ± 0.90	0.87 ± 0.50	1.49 ± 1.00	0.076
CD45+ cell, 1000/μL	343.05 ± 100.35	297.66 ± 103.38	366.74 ± 92.24	0.082
Troponin-I after cell therapy	1.24 ± 3.30	1.02 ± 2.64	1.42 ± 3.78	0.430
Clinical presentation				
CCS angina score at baseline	2.55 ± 0.75	2.27 ± 0.64	2.80 ± 0.58	0.003
CCS angina score ≥ 3, *n* (%)	36 (55.4%)	11 (36.7%)	25 (71.4%)	0.005
CCS angina score at 3 months	0.57 ± 0.77	0.33 ± 0.55	0.77 ± 0.88	0.021
CCS angina score at 6 months	0.50 ± 0.74	0.38 ± 0.62	0.60 ± 0.66	0.282
CCS angina score at 12 months	0.33 ± 0.57	0.17 ± 0.47	0.47 ± 0.62	0.021
NYHA Fc of dyspnea at baseline	1.71 ± 1.13	1.43 ± 1.17	1.94 ± 1.06	0.086
NYHA Fc of dyspnea at 3 months	0.71 ± 0.88	0.37 ± 0.62	1.00 ± 0.97	0.003
NYHA Fc of dyspnea at 6 months	0.53 ± 0.87	0.17 ± 0.47	0.83 ± 1.01	0.001
NYHA Fc of dyspnea at 12 months	0.54 ± 0.90	0.21 ± 0.56	0.82 ± 1.03	0.002
Examination				
Endothelial dysfunction*, *n* (%)	38 (64.4%)	15 (57.7%)	23 (69.7%)	0.339
CPET METs at baseline	5.08 ± 1.17	4.83 ± 1.19	5.24 ± 1.16	0.255
CPET METs at 6 months	5.33 ± 1.44	5.22 ± 1.44	5.38 ± 1.46	0.750
Difference of METs ^6 months vs. baseline^	0.17 ± 1.20	0.47 ± 0.86	0.01 ± 1.33	0.252
CMR LVEF at baseline, %	50.31 ± 14.52	52.69 ± 13.28	48.21 ± 15.42	0.214
CMR LVEF at 6 months, %	52.93 ± 14.08	56.25 ± 12.33	50.03 ± 15.05	0.098
Difference of LVEF^6 months vs. baseline^	2.02 ± 4.96	2.71 ± 3.87	1.41 ± 5.74	0.312
Angiogenesis score at baseline	1.57 ± 0.63	1.73 ± 0.57	1.43 ± 0.66	0.048
Angiogenesis score ≥ 2, *n* (%)	36 (56.3%)	20 (66.7%)	16 (47.1%)	0.115
Angiogenesis score at 9 months	2.60 ± 0.83	2.65 ± 0.70	2.56 ± 0.94	0.955
3D echo LVEF at baseline, %	51.30 ± 10.62	49.41 ± 10.38	52.91 ± 10.71	0.188
3D echo LVEF ≥ 50%, *n* (%)	40 (61.5%)	17 (56.7%)	23 (65.7%)	0.455
3D echo LVEF ≤ 40%, *n* (%)	9 (13.8%)	6 (20.0%)	3 (8.6%)	0.282
3D echo LVEF at 3 months, %	55.01 ± 11.46	56.18 ± 11.36	54.11 ± 11.63	0.494
Difference of 3D LVEF^3 months vs. baseline^	3.24 ± 5.91	5.71 ± 5.60	1.35 ± 5.49	0.002
3D echo LVEF at 6 months, %	56.45 ± 12.56	57.40 ± 13.74	55.67 ± 11.64	0.359
Difference of 3D LVEF^6 months vs. baseline^	4.89 ± 7.94	7.47 ± 10.06	2.76 ± 4.80	< 0.001
Baseline echocardiography				
LA diameter, mm	42.17 ± 5.39	42.70 ± 4.94	41.71 ± 5.79	0.369
LVEDD, mm	53.25 ± 8.42	53.22 ± 8.37	53.27 ± 8.59	0.981
2D echo LVEF, %	54.32 ± 13.03	53.85 ± 13.82	54.72 ± 12.51	0.790
Grade 2 or 3 diast. dysfxn, *n* (%)	26 (40.6%)	7 (23.3%)	19 (55.9%)	0.008
TVPG, mmHg	18.32 ± 11.66	19.52 ± 13.16	17.31 ± 10.33	0.568
Moderate to severe MR, *n* (%)	9 (13.8%)	5 (16.7%)	4 (11.4%)	0.722
3D echo LVEDV, mm^3^	87.11 ± 27.14	86.60 ± 26.43	87.51 ± 28.08	0.896
Systolic dyssynchrony index, %	7.75 ± 7.25	8.48 ± 8.81	7.10 ± 5.54	0.850
Outcome at 1 year				
Composite endpoints^†^, *n* (%)	31 (47.7%)	14 (46.7%)	17 (48.6%)	0.878
All-cause mortality, *n* (%)	10 (15.4%)	4 (13.3%)	6 (17.1%)	0.742
Cardiovascular death, *n* (%)	1 (1.5%)	1 (3.3%)	0 (0.0%)	0.462
Acute myocardial infarction, *n* (%)	3 (4.6%)	1 (3.3%)	2 (5.7%)	1.000
Hospitalization for HF, *n* (%)	13 (20.0%)	6 (20.0%)	7 (20.0%)	1.000
Revascularization, *n* (%)	16 (24.6%)	9 (30.0%)	7 (20.0%)	0.351
Sepsis, *n* (%)	6 (12.3%)	2 (6.7%)	6 (17.1%)	0.270

Notes: Responder was defined as 1-year improvement of LVEF ≥ 7.0% after cell-based therapy for EnD-CAD. Data are expressed as mean ± standard deviation or number (percentage)

*Abbreviation*: *GCSF* granulocyte-colony stimulating factor, *CV* coefficient of variability, *CCS* Canadian Cardiovascular Society, *NYHA Fc* New York Heart Association functional classification, *CPET* cardiopulmonary exercise testing, *MET* metabolic equivalent of task, *CMR* cardiovascular magnetic resonance imaging, *LVEF* left ventricular ejection fraction, *3D echo* three-dimensional echocardiography, *2D echo* two-dimensional echocardiography, *LA* left atrium, *LVEDD* left ventricular end-diastolic diameter, *diast. dysfxn* diastolic dysfunction, *TVPG* trans-tricuspid valve pressure gradient, *MR* mitral regurgitation, *LVEDV* left ventricular end-diastolic volume, *HF* heart failure

*Endothelial dysfunction was defined as post-nitroglycerin flow-mediated dilatation of brachial artery < 300%

^†^Composite endpoints were comprised of all-cause mortality, major adverse cardiac or cerebrovascular events (defined as cardiovascular death, acute myocardial infarction, or stroke), hospitalization for heart failure, or unexpected revascularization

The responders had significantly lower angina severity and insignificantly less dyspnea compared with the non-responders prior to CD34+ cell therapy. Within the follow-up period, the responders had better clinical symptomatic improvement in both angina and HF as compared with the non-responders at the time points of every 3 months, indicating the improvement of cardiac systolic function (i.e., LVEF improvement ≥ 7.0%) was correlated with the relief of clinical symptoms.

Regarding objective evaluations for functional capacity, angiogenesis, chamber sizes, and cardiac/valvular functions, there were no significant differences between groups at baseline. However, the responders had higher coronary angiogenesis score and less echocardiographic grade 2 or 3 diastolic dysfunction than the non-responders. Notably, the difference of LVEF on 3D echocardiography between the follow-up period and baseline began to be significant at 3 months after stem cell therapy, implicating good clinical, and subclinical responses could be observed as early as 3 months since delivery of CD34+ cells. After 1-year follow-up, composite endpoints occurred in nearly one half of EnD-CAD patients but did not differ between the two groups. Around 1 in 5 patients needed hospitalization for acute decompensated HF, and nearly 1 in 4 patients received salvage myocardial revascularization strategy for relief of refractory angina in both groups.

### Identification of “predictors of good responder” to CD34+ cell therapy from baseline characteristics or presentations (Table 3, Figs. 1 and 2)

To understand which baseline variable could be predictive of a good responder prior to CD34+ cell therapy in patients with EnD-CAD, logistic regression analysis was performed. In univariate analysis, male gender, higher baseline leukocyte count, CCS angina score ≥ 3, and grade of diastolic dysfunction ≥ 2 were identified as potentially poor responders to the cell therapy. On the contrary, a former smoker and higher baseline angiogenesis score could be used to predict good response to the cell therapy. After multivariate adjustment for the above potential variables, the “presence” of the former smoker and “absence” of the male gender, Canadian Cardiovascular Society (CCS) angina score ≥ 3, and grade of diastolic dysfunction ≥ 2 on the initial survey were identified as independent predictors of good responder after stem cell therapy.

**Table 3:** Baseline predictors of “good responder” before IC CD34+ therapy for EnD-CAD

LVEF improvement ≥ 7.0%	Univariate analysis			Multivariate analysis		
Variables	OR	95% CI	*P* value	OR	95% CI	*P* value
Baseline characteristics						
Age per year	1.027	0.967–1.090	0.389			
Age ≥ 65 years	1.524	0.571–4.065	0.400			
Male sex	0.301	0.082–1.106	0.071	0.028	0.003–0.261	0.002
Body mass index	0.985	0.879–1.104	0.799			
Former smoker	2.857	1.024–7.970	0.045	8.898	1.891–41.858	0.006
Diabetes mellitus	1.102	0.357–3.405	0.866			
Atherosclerotic risk factor*	n/a	n/a	n/a			
Old stroke or old MI	0.706	0.263–1.893	0.489			
Chronic hepatitis B or C	0.267	0.028–2.533	0.250			
History of CABG	0.406	0.138–1.194	0.101			
Left main involvement	0.583	0.195–1.747	0.335			
CTO at LAD	0.962	0.362–2.560	0.939			
Leukocyte count	0.765	0.576–1.018	0.066	0.860	0.632–1.170	0.077
Hemoglobin	0.950	0.729–1.237	0.702			
Serum creatinine	0.776	0.292–2.060	0.610			
eGFR	1.003	0.981–1.027	0.766			
Total Cholesterol	1.004	0.992–1.016	0.528			
Beta blocker	n/a	n/a	n/a			
RAS inhibitor	0.424	0.111–1.622	0.210			
Lipid lowering agent	0.800	0.278–2.299	0.679			
Presentation and exams at baseline						
CCS angina score ≥ 3	0.232	0.082–0.658	0.006	0.138	0.033–0.576	0.007
NYHA Fc of dyspnea ≥ 3	0.664	0.220–2.009	0.468			
Endothelial dysfunction	0.593	0.202–1.738	0.341			
CPET-METs	0.732	0.429–1.246	0.250			
CMR LVEF	1.022	0.987–1.059	0.225			
Angiogenesis score ≥ 2	2.250	0.816–6.207	0.117			
3D echo LVEF	0.968	0.923–1.106	0.187			
3D echo LVEF ≥ 50%	0.682	0.250–1.863	0.456			
3D echo LVEF ≤ 40%	2.667	0.605–11.756	0.195			
Baseline echocardiography						
LA diameter	1.035	0.944–1.136	0.461			
LVEDD	0.999	0.943–1.059	0.980			
2D echo LVEF	0.995	0.958–1.033	0.786			
Grade 2 or 3 diast. dysfxn	0.240	0.081–0.710	0.010	0.104	0.022–0.505	0.005
TVPG	1.017	0.972–1.064	0.469			
Moderate to severe MR	1.550	0.376–6.390	0.544			
3D echo LVEDV	0.999	0.980–1.017	0.894			
Systolic dyssynchrony index	1.028	0.955–1.106	0.466			

Notes: *Abbreviation*: *IC* intracoronary, *EnD-CAD* end-stage diffuse coronary artery disease, *LVEF* left ventricular ejection fraction, *OR* odds ratio, *CI* confidence interval, *n/a* not applicable, *MI* myocardial infarction, *CABG* coronary artery bypass grafting surgery, *CTO* chronic total occlusion, *LAD* left anterior descending artery, *eGFR* estimated glomerular filtration rate, *RAS* renin-angiotensin system, *CCS* Canadian Cardiovascular Society, *NYHA Fc* New York Heart Association functional classification, *CPET* cardiopulmonary exercise testing, *MET* metabolic equivalent of task, *CMR* Cardiovascular magnetic resonance imaging, *LVEF* left ventricular ejection fraction, *3D echo* three-dimensional echocardiography, *LA* left atrium, *LVEDD* left ventricular end-diastolic diameter, *2D echo* two-dimensional echocardiography, *diast. dysfxn* diastolic dysfunction, *TVPG* trans-tricuspid valve pressure gradient, *MR* mitral regurgitation, *LVEDV* left ventricular end-diastolic volume

*Atherosclerotic risk factors indicate hypertension, diabetes, or dyslipidemia; data was not available because all EnD-CAD patients had at least one of the atherosclerotic risk factors

**Fig. 1:**
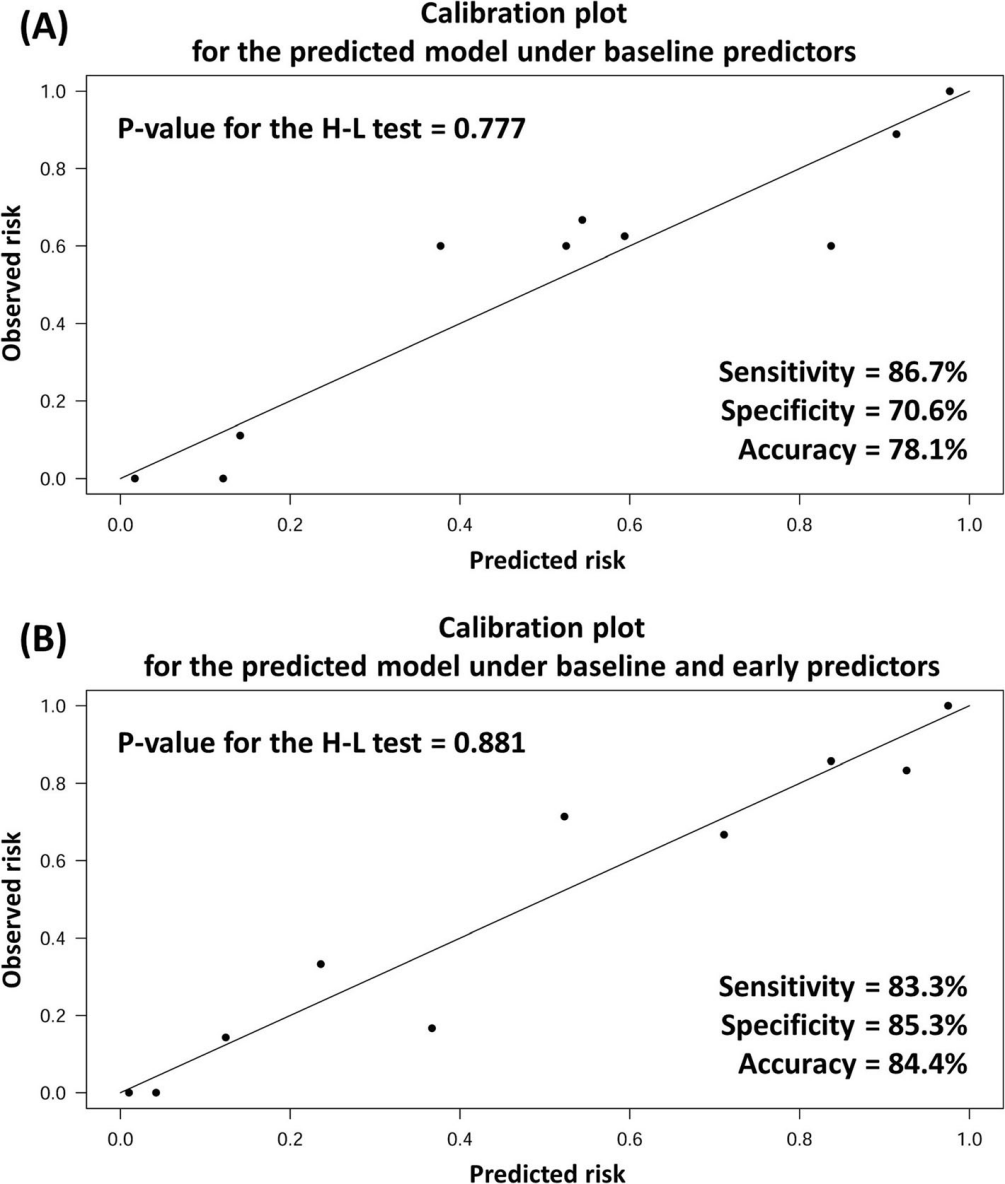
Hosmer–Lemeshow (H-L) test for goodness of fit in the logistic regression model. **a** Sensitivity 86.7%, specificity 70.6%, and accuracy 78.1% were available after the adoption of logistic regression analysis with multivariate adjustment for the male sex, past smoker, baseline CCS angina score ≥ 3, and grade 2/3 diastolic dysfunction. *P* value for the goodness of fit with H-L test was 0.777 when cutoff point was set as 0.5. **b** After adding the variable of post-G-CSF neutrophil count to the aforementioned four variables, sensitivity 83.3%, specificity 85.3%, and accuracy 84.4% were obtained for a better prediction. The *P* value for H-L test was 0.881 (cutoff point 0.5). Abbreviation: CCS = Canadian Cardiovascular Society; G-CSF = granulocyte-colony stimulating factor

**Fig. 2:**
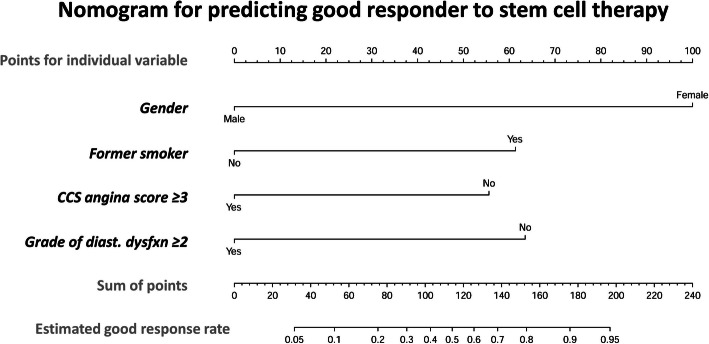
Nomogram for scaling a good response rate to stem cell therapy for EnD-CAD. The nomogram was designed for scaling and calculating the probability of good response rate to cell therapy for patients with EnD-CAD by using individual baseline variable. The scales could help investigators to calculate the response rate and further predict who will be a “good responder” before applying cell infusion to treat EnD-CAD or refractory angina. Abbreviation: EnD-CAD = end-stage diffuse coronary artery disease; CCS = Canadian Cardiovascular Society; diast. Dysfxn = diastolic dysfunction

H-L test shown in Fig. 1a demonstrated sensitivity 86.7%, specificity 70.6%, and accuracy 78.1% after considering the above four predictors (*P* = 0.777, which was higher than the cutoff value of 0.5). To facilitate an efficient evaluation in clinical practice, the nomogram in Fig. 2 was utilized to calculate the estimated good response rate of CD34+ cell therapy for EnD-CAD. Summation of individual points available from gender, former smoker, CCS angina score, and grade of diastolic dysfunction helps researchers to assess the probability of good responder when EnD-CAD patients are enrolled as candidates for cell therapy.

### Identification of “early predictors of good responder” after receiving G-CSF injection or CD34+ cell therapy (Table 4, Fig. 1 and Supplemental Table 1)

Owing to a lot of useful information available after administering G-CSF or transfusing CD34+ cells, those variables viable to be recognized as “early predictors” of a good responder, e.g., the vasculogenic activity of stem cells, biomarkers before and after G-CSF injection, and detailed imaging for angiogenesis (refer to Supplemental Table 1), were collected and added to the logistic regression analysis. Table 4 shows in addition to the four aforementioned baseline characteristics, the elevation of post G-CSF-treated neutrophil count was found negatively associated with good response to the cell therapy after multivariate adjustment. After adding this newly-identified independent predictor to the H-L test, a better predictor power was obtained with sensitivity 83.3%, specificity 85.3%, and accuracy 84.4% (*p* = 0.881, which was much higher than the cutoff value of 0.5) (Fig. 1b). Regrettably, we did not further identify other potential predictors of good responder from remaining variables after G-CSF, prior to, or after stem cell therapy.

**Table 4:** Early predictors of “good responder” after receiving G-CSF injection or stem cell therapy

LVEF improvement ≥ 7.0%	Univariate analysis			Multivariate analysis		
Variables	OR	95% CI	*P* value	OR	95% CI	*P* value
Baseline characteristics						
Male sex	0.301	0.082–1.106	0.071	0.014	0.001–0.182	0.001
Former smoker	2.857	1.024–7.970	0.045	11.032	2.028–60.004	0.005
CCS angina score ≥ 3	0.232	0.082–0.658	0.006	0.092	0.019–0.449	0.003
Grade 2 or 3 diast. Dysfxn	0.240	0.081–0.710	0.010	0.075	0.012–0.477	0.006
Post G-CSF biomarkers						
Post G-CSF leukocyte count, μL	0.952	0.905–1.002	0.058	n/a	n/a	0.906
Post G-CSF HPC count, μL	0.987	0.972–1.002	0.080	n/a	n/a	0.208
Post G-CSF young cell count	0.992	0.900–1.093	0.869			
Post G-CSF neutrophil count	0.953	0.901–1.009	0.097	0.905	0.831–0.986	0.022
Flow data: stem cell, %	0.155	0.014–1.702	0.127			
Performance CV, %	0.943	0.783–1.135	0.533			
CD34+ cell, 1000/μL	0.350	0.111–1.098	0.072			
CD45+ cell, 1000/μL	0.992	0.984–1.001	0.068			
Troponin-I after cell therapy	0.962	0.816–1.134	0.641			
Biomarkers on ELISA						
VEGF after G-CSF	1.002	1.000–1.004	0.082			
ANP-1 after G-CSF	1.003	0.997–1.009	0.352			
EGF after G-CSF	0.996	0.991–1.001	0.137			
HGF after G-CSF	1.000	1.000–1.000	0.462			
TGF-β1 after G-CSF	0.986	0.956–1.016	0.359			
SDF-1α after G-CSF	1.000	1.000–1.000	0.123			
SDF-1α before SCT	1.000	0.999–1.000	0.057			
Change of Matrigel assay before and after G-CSF injection						
Total tube length	1.000	0.999–1.000	0.118			
Mean tube length	0.990	0.978–1.003	0.125			
Number of tube formation	0.969	0.905–1.037	0.363			
Number of cluster formation	1.054	0.978–1.137	0.167			
Number of network formation	0.893	0.771–1.035	0.132			

Notes: *Abbreviation*: *G-CSF* granulocyte-colony stimulating factor, *LVEF* left ventricular ejection fraction, *OR* odds ratio, *CI* confidence interval, *n/a* not applicable, *MI* myocardial infarction, *CCS* Canadian Cardiovascular Society, *diast. dysfxn* diastolic dysfunction, *WBC* white blood cell, *HPC* hematopoietic progenitor cell, *CV* coefficient of variability, *ELISA* The enzyme-linked immunosorbent assay, *VEGF* vascular endothelial growth factor, *ANP* atrial natriuretic peptide, *EGF* epidermal growth factor, *HGF* hepatocyte growth factor, *TGF-β1* transforming growth factor beta 1, *SDF-1α* Stromal cell-derived factor-1 alpha, *SCT* stem cell therapy

As shown in Supplemental Table 1, the responders had significantly higher soluble angiogenesis levels of vascular endothelial growth factor (VEGF) and hepatocytes growth factor (HGF) in circulation as compared with the non-responders. On the other hand, the circulatory level of stromal cell-derived factor (SDF)-1α, another angiogenic/proinflammatory factor, was consistently lower in the responders than in the non-responders prior to and after G-CSF injections as well as after CD34+ cell therapy. Additionally, SDF-1α levels in coronary sinus checked at different time points were also significantly lower in the former group than in the latter one. However, coronary angiogenesis on Wimasis analysis, angiogenic capacity on Matrigel assay, and levels of angiopoietin (ANP)-1, epidermal growth factor (EGF), and transforming growth factor (TGF)-β1 (i.e., three soluble angiogenesis factors) on enzyme-linked immunosorbent assay (ELISA) did not differ between the two groups, suggesting prediction of a good responder to cell therapy was mainly dependent on baseline characteristics rather than on those variables collected after G-CSF or CD34+ cell therapy. These findings were very useful and practical on the screening for potential good responders to the cell therapy in the early stage of the trial.

## Discussion

This study which utilized and analyzed the parameters from our phase I/II clinical trials delineated several fundamental clinical-relevant information. First, the baseline variables of female gender and former smoker were significantly and positively predictive of, whereas the advanced angina score (i.e., CCS angina score ≥ 3) and moderate to severe LV diastolic dysfunction (i.e., grade ≥ 2) were negatively predictive of, good response to CD34+ cell therapy for EnD-CAD. Second, increased neutrophil count after G-CSF treatment not only was negatively predictive of good response to the cell therapy but also augmented the predictive power when it was considered in addition to the four aforementioned baseline variables. Third, most predictors of a good responder were identified based on patient’s baseline characteristics and laboratory/examination findings, suggesting that we are able to expect the probability of effectiveness and responsiveness before conducting expensive cell-based therapy.

It is well recognized that regenerative medicine is currently of paramount importance for organ dysfunction, especially for those patients with ischemia-related LV dysfunction. Abundant data [11–16, 18–20] have also supported that cell therapy is an alternative to conventional anti-ischemic treatment for the patients with intractable ischemic cardiomyopathy, complex diffuse coronary stenotic lesions, and refractory angina. However, not all the patients with EnD-CAD who received cell therapy had satisfactory clinical outcomes and prominent improvement of LV function. This issue drives the investigators [13, 22, 23] trying to integrate the consensus of improvement of LVEF ≥ 5.0 to 7.0% to be recognized as the “acceptable or great response” after cell therapy. Surprisingly, how many (i.e., percentage) or what kind of EnD-CAD patients met the criteria of “great response” to cell therapy have not yet been identified. One novel finding in the present study was that the analytical results of pooled data from our phases I/II trials showed that nearly 50% of CD34+ cell treated patients met the criteria of “good responders”, i.e., LVEF improved ≥ 7.0%. Our finding, in addition to extending the findings of the previous studies, [13, 22, 23] is the first one to highlight the early prediction of “good responders” and certainly would provide useful information in future clinical cell-based researches for the treatment of refractory angina or EnD-CAD.

Despite the important issue of good response after cell therapy has been extensively investigated and 5.0 to 7.0% of improvement in LVEF is viewed as a well acceptable value [13, 22, 23], so far, no study has reported which variables could be served as independent predictors of good response to cell-based therapy in the field of ischemic heart disease. In fact, there is only one similar study [25] which identified age, blood fibrinogen, arterial occlusion level, transcutaneous pressure of oxygen, and the transplanted CD34+ cell count were significant prognostic factors of the responders in patients with no-option critical limb ischemia. Somewhat different from the analytic results in the former study [25], our findings in the present study emphasized the importance of baseline patients’ characteristics rather than laboratory variables or lesion complexities. As for the patients with EnD-CAD, we successfully identified that the baseline variables of female gender and former smoker were independently predictive of good responders of CD34+ cell therapy, whereas the advanced angina score and diastolic dysfunction were two independent predictors of non-responders of CD34+ cell therapy. Of distinctive finding was that an increase in the neutrophil count after G-CSF treatment was another negative predictor of non-responder to cell therapy. Our findings, therefore, provide extremely useful research information on the screening and enrollment of suitable candidates for receiving CD34+ cell therapy, especially for those EnD-CAD patients who are refractory to conventional therapy.

We found discrepancies among improvements of LVEF, presentation of HF symptoms, and unfavorable clinical outcomes. In many clinical circumstances, LVEF is not consistent with the New York Heart Association functional class because the latter is namely dependent on patient’s physiological adaptation, metabolic status, oxygen consumption, and myocardial oxygen balance determined by the ratio of oxygen supply to oxygen demand [26]. Therefore, besides N-terminal pro-brain natriuretic peptide level, LVEF, 6-min walk distances, and CPET, well-designed questionnaires are suggested for functional evaluation and risk stratification in HF patients [27].

There is a close association between an increase in the circulatory level of soluble angiogenesis factors and enhancement of microvasculature/microcirculation, resulting in an improvement of LVEF [11, 12]. In the present study, circulating angiogenesis factors, such as VEGF and HGF, were also notably higher in the responders than in the non-responders (refer to Supplemental Table 1). Accordingly, our findings were consistent with those of our previous studies [11, 12].

SDF-1α, a chemokine protein of CXCL12, not only plays a beneficial role in the mobilization of EPCs from the bone marrow to circulation and homing of EPC to the ischemic area for angiogenesis through CXCR4-dependent mechanism [28, 29], but also is associated with an increased risk of CAD via leukocyte activation and proinflammatory stimulation [30]. An interesting finding of ELISA in the present study was that the levels of SDF-1α in the circulation and coronary sinus at different time points were consistently and notably lower in the responders than in the non-responders (refer to Supplemental Table 1), suggesting the non-responders presented with more severe coronary lesions (i.e., higher rates of old MI, CABG history, left main involvement and CTO at LAD shown in Table 1) and a more dominant intrinsic response to ischemic stimulation (i.e., higher levels of SDF-1α) than the responders.

Regrettably, this study did not measure the circulatory level of thrombospondin-1 (TSP1), a natural inhibitor of neovascularization, notably increased in the platelet-poor plasma of aged adults, and those with cardiovascular and metabolic disease [31]. Additionally, TSP1 has been identified to inhibit endothelial function, stimulates apoptosis, and suppresses nitric oxide, vascular endothelial growth factor, and the pluripotent transcription factors Oct3/4, Sox2, Klf4, and cMyc signaling [32]. These aforementioned issues raise the hypothesis that the low circulatory TSP1 level, perhaps, could be an extremely useful biomarker for predictive of responder and good outcome in our study.

The other interesting finding of the present study was former smokers closely linked to a good responder of stem cell therapy in the EnD-CAD patients. This association was compatible with the previous misleading phenomenon of “smoker’s paradox” [33, 34], indicating better clinical outcomes were observed among the smokers suffering from acute myocardial infarction. Recent clinical researches [35, 36] have debunked that bizarre “smoker’s paradox” was mainly affected by age factor because the majority of smokers presenting to the hospital were 10 years younger than nonsmokers. In addition, further analysis in our study revealed that the former smokers not only were younger, taller, and heavier but also had better eGFR and less history of CABG (refer to Supplemental Table 2). As a result, our findings and recent deep investigations could be explained, at least in part, for the close relationship of “former smoker” to “good responder” of cell therapy.

In the current study, 20% of patients in both groups suffered from acute decompensated HF with a need for hospitalization for further management. If there had been no evidence of myocardial injury, i.e., an elevated level of cardiac troponin-I, the patient was discharged without CAG after HF symptoms were improved. Otherwise, those with elevated troponin-I would undergo CAG and receive bailout coronary stenting at culprit lesion to increase myocardial perfusion. By the 9th month, all study subjects received follow-up CAG for the assessment of angiogenesis. Majority of patients in both groups underwent diagnostic CAG only. However, if the coronary flow had been limited by critical or progressive stenotic lesions as compared with baseline CAG findings as well as the vessel larger and suitable for intervention after cell therapy, myocardial revascularization with PCI would be performed as a bailout therapeutic strategy.

This study has limitations. First, despite the four variables were identified to be strongly predictive of either good responder or non-responder in the present study, these variables were not valid factors predictive of 1-year untoward clinical outcomes (refer to Supplemental Table 3) after CD34+ cell therapy. A prospective study to test predictors of good outcomes such as longer survival or better functional/echocardiographic improvement is encouraged. However, there are still several issues to be addressed in a prospective case-control study that will be conducted, e.g., difficulty in patient’s enrollment and high event rate in such high-risk EnD-CAD patients. Maybe, a retrospective study with a longer follow-up period or increase in study case number would be a better solution to answer the question. At that time, we will be able to apply cell-based therapy in highly selected patients or specific patient population with cost-effectiveness. Second, the long-term clinical follow-up (i.e., more than 1 to 5 years follow-up) is continuously conducted in phase II clinical trial. Accordingly, whether the currently identified variables will be able to predict 5-year “good responder” or “non-responder” remains to be answered.

## Conclusions

The results of this study identified four baseline variables for the prediction of good responders prior to CD34+ therapy for the patients with EnD-CAD. These findings may provide essential and useful information on screening suitable candidates in a clinical cell-based research.

## Supplementary information

**Additional file 1 : Table S1**. Angiogenesis, biomarkers, and cell migration function before and after stem cell therapy.

**Additional file 2 : Table S2.** Variables of interest compared between smokers versus non-smokers.

**Additional file 3 : Table S3**. To test whether the new 4 identified factors can be used to predict adverse clinical events.

## Data Availability

The datasets generated and analyzed are not publicly available due to consideration of patient privacy, but are available from the corresponding author on reasonable request for academic purpose.

## Abbreviations

PCI: Percutaneous coronary intervention
CABG: Coronary artery bypass grafting surgery
CAD: Coronary artery disease
EnD-CAD: End-stage diffuse coronary artery disease
EPC: Endothelial progenitor cell
MSC: Mesenchymal stem cell
LV: Left ventricular
LVEF: Left ventricular ejection fraction
G-CSF: Granulocyte-colony stimulating factor
H-L test: Hosmer–Lemeshow test
CTO: Chronic total occlusion
LAD: Left anterior descending
MI: Myocardial infarction
eGFR: Estimated glomerulus filtration rate
CCS: Canadian Cardiovascular Society
VEGF: Vascular endothelial growth factor
HGF: Hepatocyte growth factor
SDF-1α: Stromal cell-derived factor-1 alpha
ANP: Atrial natriuretic peptide
EGF: Epidermal growth factor
TGF: Transforming growth factor
ELISA: The enzyme-linked immunosorbent assay
