# Supplementary material for: Baseline factors identified for the prediction of good responders in patients with end-stage diffuse coronary artery disease undergoing intracoronary CD34+ cell therapy
Source: Stem Cell Res Ther. 2020 Jul 29;11:324. doi: 10.1186/s13287-020-01835-z (PMC7391819; doi:10.1186/s13287-020-01835-z)
Supplement: Supplementary file 1 — Additional file 1 : Table S1. Angiogenesis, biomarkers, and cell migration function before and after stem cell therapy. [file 13287_2020_1835_MOESM1_ESM.docx]

| **Table S1**. Angiogenesis, biomarkers, and cell migration function before and after stem cell therapy | | | | |
| --- | --- | --- | --- | --- |
| Variable | All  (N = 65) | Responder  (N = 30) | Non-responder  (N = 35) | p-value |
| CAG imaging for angiogenesis |  |  |  |  |
| Wimasis analysis at baseline |  |  |  |  |
| Vessel density, % | 23.80±9.20 | 21.32±8.48 | 25.56±9.43 | 0.117 |
| Total vessel network, 10^3^ pixel | 11.15±5.47 | 9.74±5.05 | 12.16±5.62 | 0.132 |
| Total segments, pixel | 813.39±603.18 | 666.13±486.86 | 918.57±662.57 | 0.174 |
| Number of total branching points | 358.11±270.51 | 292.63±218.01 | 404.90±297.48 | 0.161 |
| Number of total nets | 37.44±24.37 | 30.47±20.47 | 42.43±26.03 | 0.074 |
| Wimasis analysis at 9 months |  |  |  |  |
| Vessel density, % | 31.22±5.98 | 32.42±3.82 | 30.56±6.87 | 0.265 |
| Total vessel network, 10^3^ pixel | 16.29±3.29 | 16.90±2.22 | 15.94±3.76 | 0.374 |
| Total segments, pixel | 1370.26±389.66 | 1413.77±293.15 | 1346.09±437.57 | 0.596 |
| Number of total branching points | 615.54±179.72 | 636.53±124.59 | 603.87±205.37 | 0.579 |
| Number of total nets | 51.53±16.15 | 50.58±17.24 | 52.06±15.83 | 0.779 |
| ELISA for biomarkers |  |  |  |  |
| VEGF before G-CSF, pg/mL | 223.53±265.39 | 322.48±263.17 | 134.82±238.23 | 0.005 |
| VEGF after G-CSF, pg/mL | 308.70±306.23 | 386.25±348.64 | 234.01±242.62 | 0.142 |
| ANP-1 before G-CSF, ng/L | 385.16±201.56 | 390.04±200.25 | 377.02±212.40 | 0.863 |
| ANP-1 after G-CSF, ng/L | 412.36±127.42 | 430.10±122.38 | 385.75±135.54 | 0.359 |
| EGF before G-CSF, pg/mL | 327.53±240.18 | 331.01±259.01 | 321.75±215.96 | 0.922 |
| EGF after G-CSF, pg/mL | 322.17±148.79 | 291.56±151.28 | 373.19±135.35 | 0.119 |
| HGF (10^3^) before G-CSF, ng/mL | 0.67±0.32 | 0.79±0.39 | 0.56±0.19 | 0.007 |
| HGF (10^3^) after G-CSF, ng/mL | 3.83±1.92 | 3.64±1.79 | 4.02±2.06 | 0.470 |
| TGF-β1 before G-CSF, ng/mL | 6.71±5.16 | 5.26±2.91 | 9.12±7.09 | 0.144 |
| TGF-β1 after G-CSF, ng/mL | 45.19±24.18 | 42.17±22.93 | 50.22±26.36 | 0.370 |
| SDF-1α (10^3^) before G-CSF, ng/mL | 1.93±1.80 | 1.43±1.71 | 2.39±1.79 | 0.034 |
| SDF-1α (10^3^) after G-CSF, ng/mL | 2.49±1.99 | 2.08±2.16 | 2.90±1.75 | 0.084 |
| SDF-1α (10^3^) before SCT, ng/mL | 1.90±1.58 | 1.42±1.53 | 2.25±1.51 | 0.073 |
| SDF-1α (10^3^) 5 min after SCT | 2.35±1.98 | 1.52±1.62 | 3.08±2.04 | 0.006 |
| SDF-1α (10^3^) 10 min after SCT | 2.18±1.91 | 1.40±1.56 | 2.81±1.93 | 0.007 |
| SDF-1α (10^3^) 30 min after SCT | 2.03±1.85 | 1.55±1.89 | 2.45±1.74 | 0.034 |
| SDF-1α (10^3^) all concentrated, ng/mL | 3.03±2.24 | 2.13±1.94 | 3.79±2.25 | 0.005 |
| Matrigel assay for angiogenesis |  |  |  |  |
| Before G-CSF |  |  |  |  |
| Total tube length (10^3^), µm | 0.57±0.98 | 0.90±1.37 | 0.45±0.88 | 0.245 |
| Mean tube length, µm | 65.41±79.47 | 97.70±108.79 | 54.11±66.23 | 0.223 |
| Number of tube formation | 4.66±4.75 | 5.23±4.29 | 4.46±4.99 | 0.598 |
| Number of cluster formation | 2.87±3.17 | 1.91±1.92 | 3.20±3.48 | 0.539 |
| Number of network formation | 0.71±1.77 | 0.69±1.56 | 0.72±1.87 | 0.224 |
| After G-CSF |  |  |  |  |
| Total tube length (10^3^), µm | 6.30±2.67 | 5.03±1.93 | 6.74±2.78 | 0.121 |
| Mean tube length, µm | 203.25±51.72 | 191.07±44.16 | 207.52±54.51 | 0.376 |
| Number of tube formation | 31.10±10.94 | 27.71±12.72 | 32.28±10.34 | 0.391 |
| Number of cluster formation | 14.36±12.40 | 18.63±13.90 | 12.87±11.85 | 0.438 |
| Number of network formation | 15.38±7.47 | 11.37±7.37 | 16.78±7.16 | 0.072 |
| Total concentrated value |  |  |  |  |
| Total tube length (10^3^), µm | 9.70±2.98 | 9.11±4.11 | 9.89±2.64 | 0.567 |
| Mean tube length, µm | 241.12±47.28 | 262.87±57.02 | 234.26±43.24 | 0.340 |
| Number of tube formation | 41.72±13.72 | 36.28±18.42 | 43.44±11.99 | 0.484 |
| Number of cluster formation | 22.66±22.21 | 30.80±28.91 | 20.08±19.91 | 0.679 |
| Number of network formation | 28.71±12.01 | 26.88±17.54 | 29.28±10.27 | 0.750 |
| Notes: Responder was defined as 1-year improvement of LVEF ≥7.0% after cell-based therapy for EnD-CAD.  Data are expressed as mean ± standard deviation or number (percentage). Abbreviation: *CAG*: coronary angiography; *ELISA*: The enzyme-linked immunosorbent assay; *VEGF*: vascular endothelial growth factor; *G-CSF*: granulocyte-colony stimulating factor; *ANP*: atrial natriuretic peptide; *EGF*: epidermal growth factor; *HGF*: hepatocyte growth factor; *TGF-β1*: transforming growth factor beta 1; *SDF-1α*: Stromal cell-derived factor-1 alpha; *SCT:* stem cell therapy. | | | | |
