# Supplementary material for: Baseline factors identified for the prediction of good responders in patients with end-stage diffuse coronary artery disease undergoing intracoronary CD34+ cell therapy
Source: Stem Cell Res Ther. 2020 Jul 29;11:324. doi: 10.1186/s13287-020-01835-z (PMC7391819; doi:10.1186/s13287-020-01835-z)
Supplement: Supplementary file 2 — Additional file 2 : Table S2. Variables of interest compared between smokers versus non-smokers. [file 13287_2020_1835_MOESM2_ESM.docx]

| **Table** **S2.** Variables of interest compared between smokers versus non-smokers | | | |
| --- | --- | --- | --- |
| Variable | Smoker (N = 27) | Non-smoker (N = 41) | p-value |
| Age, year | 62.44±8.92 | 66.34±7.55 | 0.057 |
| Male sex, n (%) | 27 (100%) | 27 (65.9%) | 0.001 |
| Body height (cm) | 164.92±5.93 | 160.93±8.74 | 0.030 |
| Body weight (kg) | 72.37±11.88 | 67.53±9.92 | 0.073 |
| History of CABG, n (%) | 3 (11.1%) | 20 (48.8%) | 0.001 |
| History of PCI, n (%) | 20 (74.1%) | 28 (68.3%) | 0.609 |
| Hemoglobin, g/dL | 13.87±1.72 | 12.86±1.89 | 0.029 |
| eGFR, ml/min/1.73m^2^ | 68.07±23.10 | 57.39±19.45 | 0.044 |
| Serum potassium, mEq/L | 4.43±0.36 | 4.19±0.43 | 0.022 |
| CCS angina score at baseline | 2.33±0.73 | 2.68±0.72 | 0.057 |
| CCS angina score ≥3, n (%) | 11 (40.7%) | 26 (63.4%) | 0.066 |
| NYHA Fc of dyspnea at baseline | 1.74±1.06 | 1.73±1.16 | 0.974 |
| Composite endpoints*, n (%) | 16 (59.3%) | 17 (42.5%) | 0.178 |
| MACCE, n (%) | 6 (22.2%) | 2 (5.0%) | 0.030 |
| Revascularization, n (%) | 9 (33.3%) | 7 (17.5%) | 0.136 |
| Notes:*Composite endpoints were comprised of all-cause mortality, major adverse cardiac or cerebrovascular events (MACCE, defined as cardiovascular death, acute myocardial infarction, or stroke), hospitalization for heart failure, or unexpected revascularization. Abbreviation: *CABG*: coronary artery bypass grafting surgery; *PCI*: percutaneous coronary intervention; *eGFR*: estimated glomerular filtration rate; *CCS*: Canadian Cardiovascular Society; *NYHA Fc*: New York Heart Association functional classification; *MACCE*: major adverse cardiac or cerebrovascular events | | | |
