# Supplementary material for: Baseline factors identified for the prediction of good responders in patients with end-stage diffuse coronary artery disease undergoing intracoronary CD34+ cell therapy
Source: Stem Cell Res Ther. 2020 Jul 29;11:324. doi: 10.1186/s13287-020-01835-z (PMC7391819; doi:10.1186/s13287-020-01835-z)
Supplement: Supplementary file 3 — Additional file 3 : Table S3. To test whether the new 4 identified factors can be used to predict adverse clinical events. [file 13287_2020_1835_MOESM3_ESM.docx]

| **Table S3**. To test whether the new 4 identified factors can be used to predict adverse clinical events | | | | | | |
| --- | --- | --- | --- | --- | --- | --- |
| Composite endpoints | Univariate analysis | | | Multivariate analysis | | |
| Variables | OR | 95% CI | P-value | OR | 95% CI | p-value |
| Male sex | 1.856 | 0.538-6.406 | 0.328 | 0.304 | 0.076-1.214 | 0.092 |
| Former smoker | 1.968 | 0.730-5.302 | 0.181 | 2.845 | 0.915-8.846 | 0.071 |
| Baseline CCS angina score ≥3 | 0.839 | 0.321-2.194 | 0.720 | 0.989 | 0.355-2.753 | 0.983 |
| Grade ≥2 diast. dysfxn | 0.816 | 0.305-2.185 | 0.686 | 0.819 | 0.289-2.325 | 0.708 |
| Notes: Abbreviation: *OR*: odds ratio; *CI*: confidence interval; *CCS*: Canadian Cardiovascular Society; *diast. dysfxn:* diastolic dysfunction | | | | | | |
